# Recent Developments in Hyaluronic Acid-Based Hydrogels for Cartilage Tissue Engineering Applications

**DOI:** 10.3390/polym14040839

**Published:** 2022-02-21

**Authors:** Evgenia Tsanaktsidou, Olga Kammona, Costas Kiparissides

**Affiliations:** 1 Chemical Process & Energy Resources Research Institute, Centre for Research and Technology Hellas, P.O. Box 60361, 57001 Thessaloniki, Greece; jtsanaktsidou@certh.gr (E.T.); kammona@certh.gr (O.K.); 2 Department of Chemical Engineering, Aristotle University of Thessaloniki, P.O. Box 472, 54124 Thessaloniki, Greece

**Keywords:** hyaluronic acid, cartilage, tissue engineering, injectable hydrogels, microgels, cryogels, bioinks, bioprinting

## Abstract

Articular cartilage lesions resulting from injurious impact, recurring loading, joint malalignment, etc., are very common and encompass the risk of evolving to serious cartilage diseases such as osteoarthritis. To date, cartilage injuries are typically treated via operative procedures such as autologous chondrocyte implantation (ACI), matrix-associated autologous chondrocyte implantation (MACI) and microfracture, which are characterized by low patient compliance. Accordingly, cartilage tissue engineering (CTE) has received a lot of interest. Cell-laden hydrogels are favorable candidates for cartilage repair since they resemble the native tissue environment and promote the formation of extracellular matrix. Various types of hydrogels have been developed so far for CTE applications based on both natural and synthetic biomaterials. Among these materials, hyaluronic acid (HA), a principal component of the cartilage tissue which can be easily modified and biofunctionalized, has been favored for the development of hydrogels since it interacts with cell surface receptors, supports the growth of chondrocytes and promotes the differentiation of mesenchymal stem cells to chondrocytes. The present work reviews the various types of HA-based hydrogels (e.g., in situ forming hydrogels, cryogels, microgels and three-dimensional (3D)-bioprinted hydrogel constructs) that have been used for cartilage repair, specially focusing on the results of their preclinical and clinical assessment.

## 1. Introduction

Articular cartilage is a glass-like tissue that lines the ends of articulating bones. It is characterized by its ability to tolerate heavy loads over the years, thus facilitating the motion of one bone against the other [1,2].

Articular cartilage injuries such as (osteo) chondral lesions can be the result of joint malalignment and/or injurious impact during sports activity, repeated loading, etc., and could lead to joint diseases such as arthritis [1,3]. It should be noted that about half a million cartilage injuries occur per year only in the United States of America (USA) [4]. They cause intense physical pain and they can be responsible for excessive medical costs, mobility decline, etc. [1]. Cartilage lesions are unable to self-heal, due to the fact that cartilage is an avascular, aneural tissue without a lymphatic network, exhibiting moderate chondrocyte growth and proliferation [5]. Accordingly, the maintenance of a healthy cartilage tissue is of enormous significance.

To date, depending on the size of the defect [4], cartilage injuries are treated via operative procedures (e.g., autologous chondrocyte implantation (ACI), matrix-associated autologous chondrocyte implantation (MACI), microfracture, mosaicplasty, joint debridement and drilling, tissue grafts, total and partial joint replacement, etc. [6,7]) with well-known drawbacks (e.g., ACI can possibly result in scarring, postoperative morbidity, generation of cartilage tissue with inferior biomechanical properties in comparison with the native tissue and thus at risk of breaking down and requiring total joint arthroplasty, etc.) [1]. In this respect, hydrogel-aided cartilage tissue engineering (CTE) could be considered a promising alternative solution for cartilage repair.

Hydrogels are three-dimensional, highly water-swollen networks characterized by adjustable rheological/mechanical properties, biocompatibility, biodegradability and mass-transfer ability (i.e., they facilitate the exchange of oxygen and soluble molecules) [8]. They can be formed using natural or synthetic polymeric materials, or a combination of them (i.e., hybrid or composite hydrogels). Hydrogels constitute a promising tool for CTE applications due to their tunable composition, structure, dimensions, as well as because of their ability to enhance the release of various cell types and bioactive molecules, while fulfilling the dynamic demands of the tissue repair process [5,8]. Finally, hydrogels embedded within cells can resemble the native cartilage tissue environment while promoting the formation of neocartilage tissue [9]. 

Hyaluronic acid (HA) is a natural glycosaminoglycan consisting of repeating disaccharide units (i.e., d-glucuronic acid and *N*-acetyl-d-glucosamine) that has been extensively used in CTE studies, since it can be found in abundance in cartilage tissue [5,10]. HA can be subjected to various modifications leading to the alteration of the material’s properties [11]. 

The present work aims to extensively review the various types of HA-based injectable hydrogels (e.g., in situ forming hydrogels, cryogels, microgels) and 3D-bioprinted HA hydrogel constructs that have been used for articular cartilage repair. The in situ forming injectable hydrogels comprise the majority of the developed hydrogels and are analytically presented in a tabulated form providing detailed information with regard to the molecular weight of HA, its degree of modification, its functionalization, the cross-linking method (e.g., photopolymerization, Michael-type addition, Schiff base, redox, etc.) the gelation onset time, the type (e.g., mesenchymal stem cells, chondrocytes) of encapsulated cells and the in vitro or in vivo outcome of the research. The clinical evaluation of acellular and cell-laden HA hydrogels in patients with knee osteoarthritis (OA) is also presented in a separate table. In detail, the safety and efficacy of acellular HA hydrogels and Cartistem^®^ (the only HA-based approved medicinal product for cartilage tissue regeneration [12]) are compared with typical HA-based viscosupplements and/or corticosteroids and microfracture, respectively.

The present review paper is based on a systematic study of PubMed and Google Scholar, using combinations of the following search terms: hyaluronic acid, hydrogels, cryogels, microgels, 3D-printed hydrogels, articular cartilage and tissue engineering. The search covered the time period from 1 January 2010 until today. Review and research papers addressing the in vitro and in vivo assessment of different types of HA hydrogels in CTE as well as related material addressing the clinical evaluation of HA hydrogels were thoroughly assessed and selected for inclusion.

## 2. Cartilage

Cartilage is a connective tissue that can be found in many areas of the body, such as the ear, the nose, the joints, the ribs, the throat, etc. It is classified as hyaline cartilage, elastic cartilage and fibrocartilage. All cartilage types consist of chondrocytes and ECM macromolecules [13]. Hyaline cartilage, characterized by its blue-white color, is the most plentiful type of cartilage in the body and it can be found mostly in joints (articular cartilage), but also in the trachea, nose, epiphyseal growth, etc. [14,15]. This smooth and elastic type of cartilage is mostly synthesized by collagen type II and proteoglycans, and it can withstand the compressive pressure at bone articulation sites. Elastic cartilage has a characteristic yellow color and is mainly found in the ear, nose, epiglottis and larynx. Moreover, it is surrounded by a perichondrium-like layer and it is also known for providing elasticity to pressure [15]. Fibrocartilage is usually found in tendons, ligaments and menisci, as well as between intervertebral disks and in the articular surfaces of several bones. In addition, this type of cartilage contains a huge amount of collagen type I, lacks perichondrium and is found in areas that need increased support and tensile strength [14,15]. Cartilage is characterized by resiliency, flexibility, semitransparency, toughness, resistance to compressive forces, ability to form a framework that enables the initiation of bone deposition, and efficiency to cover the joint surfaces, thus enabling bone sliding with decreased friction [14,15]. On the other hand, this connective tissue has limited regenerative ability, since it lacks nerves as well as blood and lymphatic vessels, and hence, the self-healing process of a probable injury is extremely difficult [14,15,16]. 

### 2.1. Articular Cartilage

The articular cartilage is a special type of hyaline cartilage covering the gliding surfaces of synovial joints. It is credited for the normal motion of joints, providing low-friction lubricated surfaces, and it is recognized as wear-resistant tissue [17].

#### 2.1.1. Composition

The articular cartilage tissue consists of a solid and a liquid phase. More specifically, the solid phase includes chondrocytes and the extracellular matrix (ECM), whereas the liquid phase contains interstitial water and electrolytes [13,16,17,18,19]. Chondrocytes correspond to a small fraction of the total cartilage tissue volume [16,18]. These metabolically active and highly specialized cells, which are originated from mesenchymal stem cells, are able to maintain, develop and fix the ECM. The anatomical part of the articular cartilage where they reside determines their shape, number and size (e.g., the cartilage cells in the superficial zone are smaller and smoother compared to the cells found in deeper zones in the matrix) [18]. Moreover, chondrocytes are able to recognize and react to several mechanical stimuli inside their microenvironment [16] and they are able to synthesize two basic components of the matrix, i.e., collagen and proteoglycan [20]. ECM contains several organic components such as collagen, proteoglycans, noncollagenous proteins and glycoproteins, which constitute most of the dry weight of cartilage tissue [16]. Several types of collagen (e.g., collagen type I, II, V, VI, IX, XI) can be found in articular cartilage, with collagen type II being the most plentiful one corresponding to 90–95% of the collagen in the matrix. Collagen type II contains an increased number of bound carbohydrate groups, thus permitting increased interaction with water in comparison with other collagen types [13,18]. Proteoglycans are responsible for providing compressive strength to the articular cartilage and constitute the second largest group of macromolecules in the cartilage matrix [17,18]. The most plentiful of all, aggrecan, can associate with hyaluronic acid in order to create large proteoglycan aggregates via link proteins [18]. Noncollagenous proteins and glycoproteins are considered responsible for the organization and preservation of the macromolecular structure of the ECM [18]. Finally, water corresponds to approximately 80% of the wet weight of articular cartilage and about 30% is found in the intrafibrillar area within the collagen [18]. Water provides the appropriate nutrients for joint lubrication and permits the weight-dependent tissue deformation [17]. It should be noted that the characteristic property of cartilage to withstand significant loads is based on the combination of the resistance to the friction of water flow and the water pressure inside the matrix [18]. 

#### 2.1.2. Structure: Zones and Regions

The articular cartilage is divided into the superficial, middle, deep and calcified zones. These four zones, from the surface to the subchondral bone, are characterized by diversity in their morphology, matrix synthesis, cellular, mechanical and metabolic properties. Note that, each zone contributes differently to the functional properties of articular cartilage. The superficial zone corresponds to 10–20% of the whole articular cartilage thickness and contains the largest amount of collagen, elongated chondrocytes, a small quantity of proteoglycans and increased water content [14,16]. It is known for its contribution to tensile strength, shear resistance at the time of articulation and adjustment of fluid permeability [16]. The middle zone is located between the superficial and the deep zone, thus providing a functional bridge, and represents approximately 40 to 60% of the whole cartilage volume [18]. It contains chondrocytes with a round shape, collagen fibrils randomly organized and increased proteoglycan content [14,16]. The deep zone provides increased resistance to compressive strength due to the perpendicular arrangement of collagen fibrils [18]. Moreover, this zone has the highest concentration of proteoglycans and lowest amount of water, and is separated from the calcified zone with a tidemark [14]. Finally, the calcified zone which is the deepest layer of the articular cartilage tissue contains a mixture of small chondrocytes with hydroxyapatite crystals and represents the transitional zone between the subchondral bone and the cartilage [14,16].

Apart from the zonal classification, articular cartilage is also divided into three regions, i.e., pericellular, interterritorial and territorial. Regional classification is based on the proximity to the chondrocytes, tissue composition and collagen fibril diameter and arrangement [16,18]. The pericellular matrix contains proteoglycans, collagen type VI and particularly other noncollagenous proteins [13,16]. Moreover, it is close to the cell membrane and it surrounds the cartilage cells. It should be noted that the pericellular region might be responsible for the beginning of signal transduction inside cartilage with load bearing [18]. The interterritorial matrix is composed of collagen fibrils with a characteristic large diameter [16]. It is the largest of the three matrices and it plays a significant role in the biomechanical properties of articular cartilage [18]. Finally, the territorial matrix which encloses the pericellular matrix, resides very far from the cells and it contains randomly arranged collagen fibrils [16,18].

## 3. Cartilage Tissue Engineering

**Figure 1:**
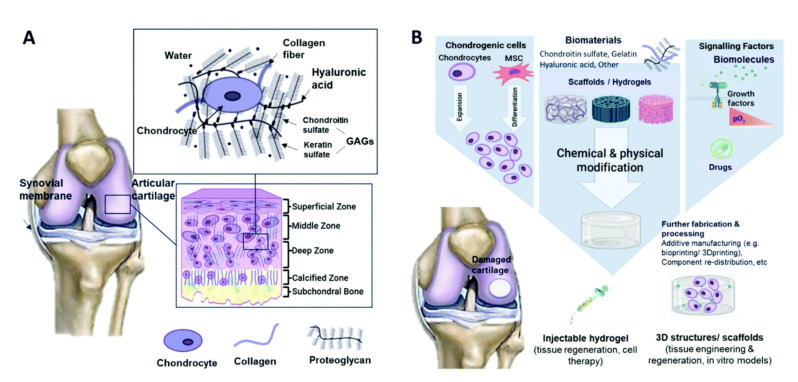
Schematic illustrations of (**A**) cartilage composition and typical tissue zones and (**B**) the tissue engineering approach for cartilage repair [22].

Tissue engineering (TE) is an interdisciplinary field applying the principles of engineering and biology towards the development of biological substitutes, which induce the restoration, maintenance or improvement of tissue function [21]. In this respect, cartilage tissue engineering (CTE) aims at the generation of biofunctional substitutes for damaged cartilage tissue (Figure 1) [3,22,23]. This rapidly evolving field involves the use of different cell types (e.g., stem cells, chondrocytes, etc.), biodegradable scaffolds made from natural or synthetic materials (e.g., sponges, membranes, injectable and noninjectable hydrogels, etc. [22]), bioactive agents (e.g., growth factors and cytokines) and physical stimuli (e.g., mechanical, electrical, etc.) [16].

An ideal scaffold for CTE should be porous, nontoxic, biocompatible/biodegradable and able to distribute nutrients. Additionally, it should favor cell differentiation and tissue formation, it should integrate with the native cartilage tissue and its degradation rate should match that of tissue formation [24]. Finally, in order to form tissues that could mimic the native ones, biomimetic scaffolds with suitable cellular responses should be developed [25].

## 4. Hydrogels—Preclinical Evaluation

Hydrogels are three-dimensional porous networks generated from cross-linked natural or synthetic polymeric chains, or their hybrids [6,26]. These extremely water-swollen networks that are able to mimic the extracellular matrix (ECM) permit the homogeneous seeding or encapsulation of different cell types, make the diffusion of solutes and nutrients possible and provide a proper environment with mechanical and chemical cues inducing cell signaling [6,10,27]. The composition, structure, mechanical and biochemical properties of these three-dimensional (3D) cross-linked networks, which are formed using hydrophilic homopolymers, copolymers, or macromers, can be easily adjusted in order to suit several biomedical applications [3,6]. More specifically, hydrogels synthesized under mild reaction conditions can be tuned regarding their rheological properties, degree of swelling, degradation and release kinetics of biochemical factors, and functionalized with cell adhesion peptides in order to feature characteristics suitable for cartilage tissue engineering (CTE) applications [10,27].

In general, hydrogels can be categorized as natural or synthetic, based on their origin, and as biodegradable or nonbiodegradable, based on their biodegradability [5]. Specifically, natural polysaccharides, such as hyaluronic acid (HA), alginate, chitosan, agarose, and protein-based materials, such as collagen, gelatin and fibrin, have acquired a lot of attention as they can be used for the development of bioactive scaffolds, exhibiting a structural resemblance to the ECM while enabling cell encapsulation and proliferation [3,5]. Hydrogels based on natural materials show adequate biocompatibility, biodegradability, a low immunoresponse and bioactive patterns encoded in their structures [6]. Furthermore, they are characterized by various degrees of compliance for supporting cell adherence and maintaining phenotype. Hydrogels based on natural polymers have evolved over the years via novel chemical and biological modifications, thus ensuring promising results in the area of CTE [8]. Table 1 shows the characteristics of the above-mentioned natural materials along with their advantages and disadvantages with respect to their application in CTE. 

Synthetic materials, such as poly(ethylene glycol) (PEG), poly(l-glutamic acid), PEG-poly(*N*-isopropyl acrylamide) (PNIPAAm), poly(vinyl alcohol) (PVA), etc., have also been used for the formation of hydrogels for CTE applications [3,5]. One of their advantages is that they can be produced in large scales with stable batch-to-batch quality [8]. Additionally, they are characterized by adjustable biodegradability, biocompatibility, mechanical and biochemical features and they are easily tunable regarding their chemical structure and molecular composition [6]. On the other hand, they are characterized by an insufficient biological activity compared to natural ones [3] and the use of potentially toxic chemicals (e.g., organic solvents, initiators, cross-linkers) in their development process. At this point it should be noted that all synthetic materials used in CTE applications should meet the physiological safety standards [8].

Apart from natural or synthetic hydrogels, composite/hybrid hydrogels are composed of two or more natural and/or synthetic polymers and combine the properties of both materials, such as biocompatibility, biodegradability and adjustable mechanical strength [6]. For example, hybrid scaffolds have been developed for CTE applications by combining natural materials such as fibrin glue, alginate and HA with synthetic polymers such as poly(lactic-co-glycolic acid) (PLGA), polyglutamic acid (PGA) and poly-ε-caprolactone (PCL) and have been found to trigger the chondrogenesis of various chondrocytes or progenitor cells [16].

### 4.1. Injectable Hydrogels

**Figure 2:**
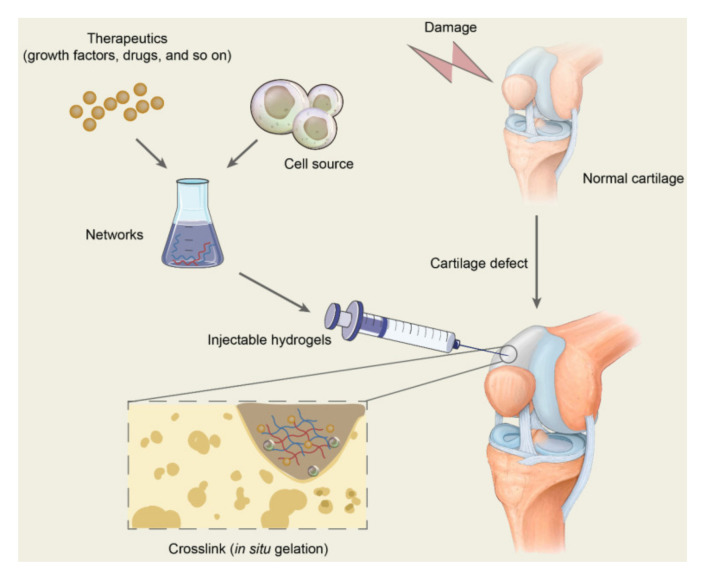
Schematic diagram of the application of injectable hydrogels for cartilage repair. Therapeutics including drugs and bioactive molecules are usually encapsulated in the networks, which are formed by polymer-based injectables. All the ingredients constitute the precursor liquid solution and are injected into the target sites of cartilage defects. The injectable hydrogels will gel in situ through chemical reactions or by physical factor induction and are expected to repair the cartilage defects [35].

Lately, injectable hydrogels have been considered attractive for tissue (e.g., cartilage, bone, skin, cardiac tissue, nerves [33]) repair. In situ formation of cell-laden biocompatible/biodegradable hydrogels, which incorporate bioactive agents, following a minimally invasive topical injection, permits the accurate filling of larger, deeper and/or irregular lesions, the spatiotemporal distribution of cells and bioactive agents and thus the enhanced targeted delivery of cells and therapeutics (e.g., growth factors, drugs, etc.) for efficient tissue growth (Figure 2) [34,35,36,37,38,39,40,41,42,43].

Apart from biocompatibility/biodegradability and nontoxicity, an ideal injectable hydrogel should meet several requirements such as gelation in aqueous media under physiological conditions (pH, temperature, ionic concentration) and at an appropriate rate for clinical application (i.e., the gelation time should be slow enough to allow accurate mixing of the constituents and prevent gelling within the needle and fast enough to prevent cells and therapeutics from settling) as well as a lack of toxic by-products [33,37,43,44,45]. Moreover, it needs to be easily administered, resemble cartilaginous ECM characteristics and stimulate the chondrogenic phenotype of cells [5].

**Figure 3:**
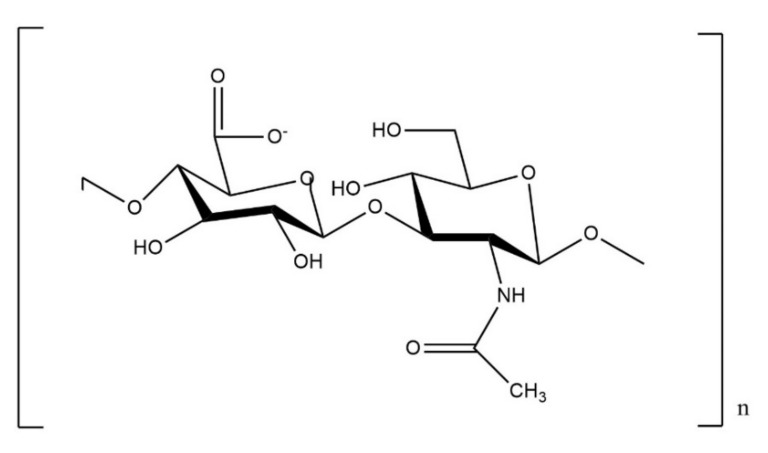
Structural formula of hyaluronic acid.

Among the natural polymers (Figures S1 and S2) used for the formation of injectable hydrogels, hyaluronic acid (HA), chondroitin sulfate (ChS), alginate, chitosan (CS) and pectin, are the ones mostly preferred [36]. HA is a linear glycosaminoglycan composed of repeating disaccharide units of d-glucuronic acid and *N*-acetyl-d-glucosamine linked by β(1,4) and β(1,3) glucosidic bonds (Figure 3) [46]. It can have 25,000 disaccharide repetitions in length with a molecular weight of 5 to 20 × 10^3^ kDa (within the joint cavity) [42]. This natural polysaccharide is present in many tissues and fluids and exists in abundance in articular cartilage, synovial fluid, dermis of the skin and vitreous of the eye [46,47]. Hyaluronan is known for supporting chondrocyte growth and MSCs differentiation towards a chondrogenic phenotype [16], and it is also well-known for its excellent viscoelastic characteristics, its increased biocompatibility and its ability to retain increased tissue hydration as well as its hygroscopic characteristics. Moreover, HA is capable of stimulating the synthesis of chondroitin-6-sulfate, type II collagen, glycosaminoglycan, hydroxyproline and DNA [6], and is known for interacting with chondrocytes through surface receptors such as CD44 and RHAMM [3]. Except for the aforementioned properties, it is important to mention that HA can be chemically modified at various sites (e.g., carboxyl, hydroxyl (primary or secondary) or *N*-acetyl groups) [48]. More specifically, hydroxyl groups can be modified by esterification and ether/hemiacetal/carbamate formation, whereas carboxyl groups can be modified by amidation, esterification, Ugi condensation and oxidation [46,49]. Finally, the modification reactions of *N*-acetyl groups involve deacetylation, hemiacetylation, hemiacetal formation and amidation [41].

The chemical modification of HA is crucial for hydrogel formation, and the properties of the newly formed hydrogels (e.g., rheological/mechanical properties, hydrophobicity, biological activity, etc.) are dependent on the type and degree of modification [11]. Accordingly, different chemical modifications of HA have been performed to various extents for cartilage tissue engineering (CTE) applications (Table 2). Additionally, already modified HA can be further functionalized using adhesion peptides such as arginylglycylaspartic acid (RGD), chondroitin sulfate binding peptide or transglutaminase substrate peptides, as well as proteins such as collagen and gelatin (Table 2) in order to enhance cell adhesion [10,31,50].

Injectable HA-based hydrogels are typically formed via chemical cross-linking (e.g., photopolymerization, click chemistry, Michael-type addition, Schiff base reaction [35,37]) (Table 2), enzymatic cross-linking (Table 2) and temperature-responsive phase transition [40]. Photocross-linking has attracted a lot of attention due to the facile control of the reaction [40]. Hydrogels are typically formed by bulk photopolymerization where the hydrogel precursor solution containing a photoinitiator is exposed to a light source [51]. However, it should be noted that the UV exposure time should be fine-tuned in order to achieve the desired mechanical properties without compromising cell viability [40].

The positive effects of HA on the cellular behavior of chondrocytes or mesenchymal stem cells (MSCs) have led to the formation of cell-laden hydrogels (Table 2) for the regeneration/reconstruction of damaged cartilage tissue. In particular, HA-based hydrogels embedded with MSCs have been shown to support the early chondrogenic differentiation of MSCs as well as the formation of neocartilage tissue in vitro and in vivo [6].

#### 4.1.1. In Situ Forming Hydrogels

**Figure 4:**
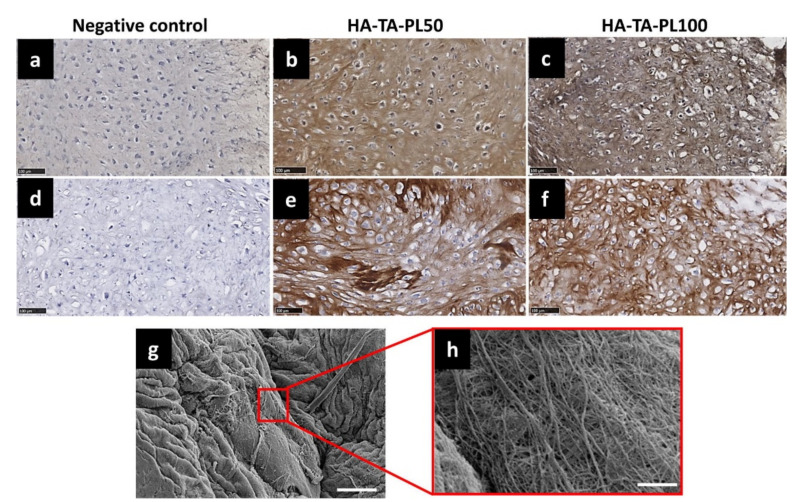
Analysis of collagen production. Immunohistochemical staining of COL II, (**a**) negative control, (**b**) HA-TA-PL50, (**c**) HA-TA-PL100 hydrogels, and COL I, (**d**) negative control, (**e**) HA-TA-PL50 and (**f**) HA-TA-PL100 hydrogels at day 28 of chondrogenesis. For the negative control, the staining procedure was performed without using a primary antibody. Scale bar is 100 µm. (**g**) SEM image of the produced collagen fibers in HA-TA-PL100 sample after 35 days of chondrogenesis. Scale bar is 10 µm, (**h**) magnification of the fibrous matrix, scale bars are 1 µm (reprinted with the permission from [52]).

Injectable, hMSC- or chondrocyte-laden HA-based hydrogels of various compositions and functional groups, forming in situ with different cross-linking methods [22], exhibiting variable gelation times and rheological/mechanical properties and encapsulating or not bioactive molecules, have been extensively tested in vitro regarding their efficacy in accumulating ECM (Table 2). In this respect, Jooybar and co-workers [52] encapsulated platelet lysate in a hMSC-laden hydrogel based on tyramine-modified hyaluronic acid (HA-TA), and examined its effect on hMSCs viability, attachment and differentiation to chondrocytes, as well as on the induction of ECM deposition. hMSCs were shown to spread and elongate in the hydrogel and differentiate to chondrocytes. Furthermore, an increased deposition of collagen type II and proteoglycans was observed over time. Finally, ECM deposition was revealed to be simultaneous with hydrogel degradation resulting in the formation of a dense and tough matrix (Figure 4). In another study, Liu and co-workers [32] developed injectable, chondrocyte-laden HA/collagen hydrogels functionalized with thiolated icariin. The formed hydrogels maintained the chondrocyte phenotype and promoted ECM secretion and fusion. Increased concentration of icariin, a flavonoid known to maintain the chondrogenic phenotype and to promote proliferation of chondrocytes, was found to enhance the formation of neocartilage ECM. Levinson and co-workers [50] loaded various amounts of transforming growth factor beta 1 (TGF-b1) (e.g., 0.25–50 ng per hydrogel) in heparin-modified HA hydrogels impregnated with chondroprogenitor cells. It was shown that a slow release of an increased concentration of TGF-b1 stimulated ECM deposition by the chondrocytes. Moreover, the developed biomaterial was proven safe for cartilage repair due to the low dose of topically administered growth factor. Thomas and co-workers [53] developed injectable hydrogels based on chitosan and oxidized HA at various ratios and studied the effect of hydrogel stiffness on the growth/functionality of the encapsulated rabbit chondrocytes. It was shown that by enhancing stiffness via the increase of the hyaluronic acid dialdehyde concentration in the hydrogel, the embedded chondrocytes maintained their spherical phenotype, exhibited a uniform distribution within the hydrogel and formed spherical aggregates. Furthermore, the expression of collagen type II and glycosaminoglycans was increased in the stiffer hydrogels in comparison with the softer ones. In another study, the differentiation of hMSCs towards a chondrogenic phenotype was found to be promoted via their encapsulation in hydrogels based on methacrylated HA (MeHA), even when they were cultured in a stem cell medium. This was further pronounced for MeHA hydrogels functionalized with a chondroitin-sulfate-binding peptide, thus denoting the positive effect of the functionalization on the expression of the chondrogenic markers collagen type II alpha 1 (Col2A1) and aggrecan (ACAN) [2]. Finally, self-cross-linking chondrocyte-laden blend hydrogels comprising thiolated HA and collagen type I were found to facilitate cell adhesion and proliferation, while the cultured rabbit chondrocytes were shown to maintain their phenotype and to secrete an abundant amount of ECM [54]. 

**Figure 5:**
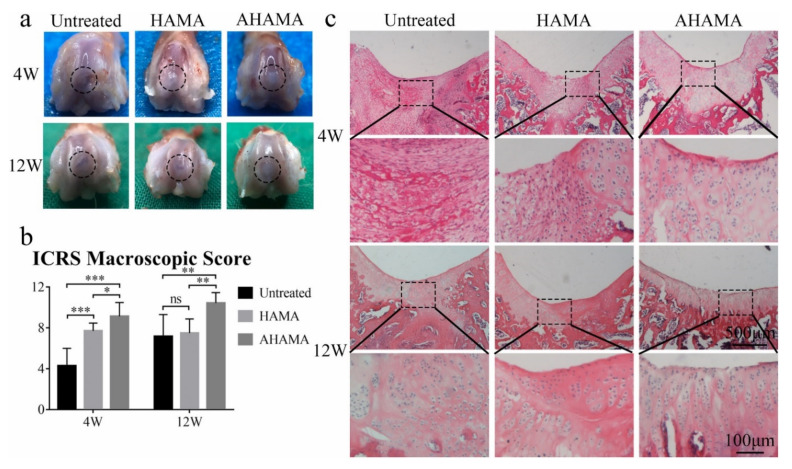
Cartilage regeneration in vivo. (**a**) Macroscopic appearance of the cartilage defect at 4 and 12 weeks post-surgery. (**b**) ICRS macroscopic scores of untreated, HAMA and AHAMA groups at 4 and 12 weeks post-surgery (*n* = 6, mean values ± SD, * *p* < 0.05, ** *p* < 0.01, *** *p* < 0.001). (**c**) H&E staining of repaired cartilage after 4 and 12 weeks post-surgery. Scale bars: up: 500 μm; down (enlarged area): 100 μm [55].

The in vivo assessment of injectable in situ forming cell-laden HA-based hydrogels and/or composite hydrogels comprising hyaluronic acid among other components has also been presented in a limited number of studies (Table 2). In a recent study, Chen and co-workers [55] developed an in situ forming hydrogel based on (aldehyde-modified) methacrylated HA. Following injection of hMSC-containing polymer solutions in rat osteochondral defects (diameter: 1.5 mm, depth: 1.5 mm) and exposure to UV light, cell-laden hydrogels were formed. The aldehyde-modified hydrogel was found to promote the integration between the native and the neocartilage tissue and thus to significantly enhance cartilage regeneration (modified O’Driscoll histological scores 12 weeks post administration: 18.3 ± 4.6) (Figure 5).

**Figure 6:**
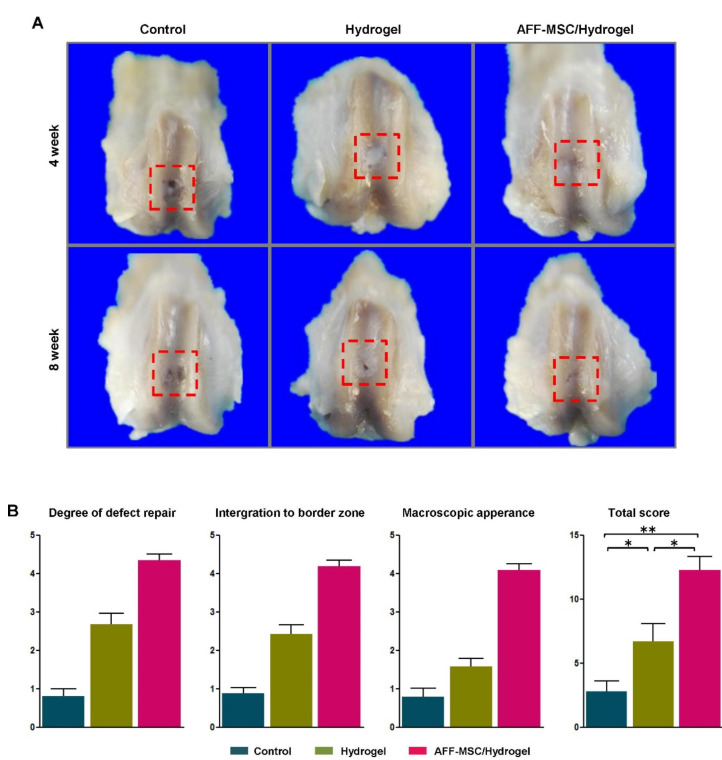
Macroscopic appearance and International Cartilage Repair Society (ICRS) quantitative score for the cartilage defect repair. (**A**) Macroscopic appearance of samples harvested at 4 and 8 weeks after surgery. (**B**) ICRS score system for gross evaluation at 8 weeks after surgery (*n* = 3). * *p* < 0.05, ** *p* < 0.01 (Reprinted with the permission from [56]).

Additionally, the topical injection of a rapidly in situ cross-linking hydrogel based on thiolated HA and hyperbranched polyethylene glycol diacrylate (polyPEGDA) and encapsulating arthroscopic flushing fluid (AFF)-derived hMSCs, was shown to promote the repair of full-thickness cartilage lesions in rats (Figure 6) [56]. Similarly, an injectable hydrogel based on tyramine-modified HA and gelatin (HTG) and embedded with porcine chondrocytes was found to promote the accumulation of ECM. Interestingly, the combination of this composite hydrogel with epigallocatechin-3-gallate (EGCG), known to suppress inflammation in various cell types including chondrocytes, resulted in cartilage protection from inflammation and diminished cartilage loss in an osteoarthritis (OA) mouse model [57]. Finally, 12 weeks after the implantation of the optimum hBMSCs-laden composite methacrylated gelatin/methacrylated HA (mGL/mHA) hydrogel in a full-length osteochondral defect in rabbits, a regeneration of cartilage and subchondral bone tissues was observed [58].

However, at this point it should be mentioned that despite the thorough assessment of various injectable hydrogels for decades, there are hardly any impeccable hydrogels for clinical application in tissue engineering. Accordingly, the development of a highly efficient injectable hydrogel for cartilage tissue engineering (CTE) is considered of outmost importance [3].

#### 4.1.2. Cryogels

**Figure 7:**
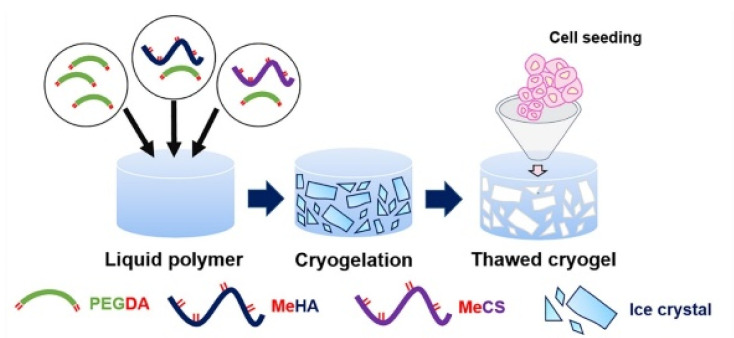
Schematic of interconnected macroporous cryogelation process: polyethylene glycol, chondroitin sulfate and hyaluronic acid were modified with methacrylate groups to enable a free radical polymerization in the frozen state through the presence of radical initiators (APS and TEMED). PEGDA, P-MeHA and P-MeCS were mixed with APS/TEMED and formed ice crystals at −20 °C for 20 h (reprinted with the permission from [84]).

Cryogels (or cryo-hydrogels) are a subcategory of hydrogels formed via physical or chemical cross-linking of natural or synthetic polymers at subzero temperatures. At these temperatures, water freezes and its crystals act as a porogen. Thawing of crystals at room temperature after gelation results in the formation of an interconnected macroporous (90% porosity [81]) structure permitting the flow of nutrients and cell trafficking thus facilitating tissue integration (Figure 7). The latter is especially important for tissues lacking blood vessels such as the articular cartilage. Cryogels are also characterized by their shape-memory properties and integrity due to their adequate degree of swelling and mechanical strength and elasticity. The use of hyaluronic acid (HA)-based cryogels as cell carriers for the repair of cartilage defects is considered promising due to their biocompatibility/biodegradability and their shape-memory properties (i.e., they contract and recover their shape after syringe injection) allowing their noninvasive topical administration via injection [81,82,83].

**Figure 8:**
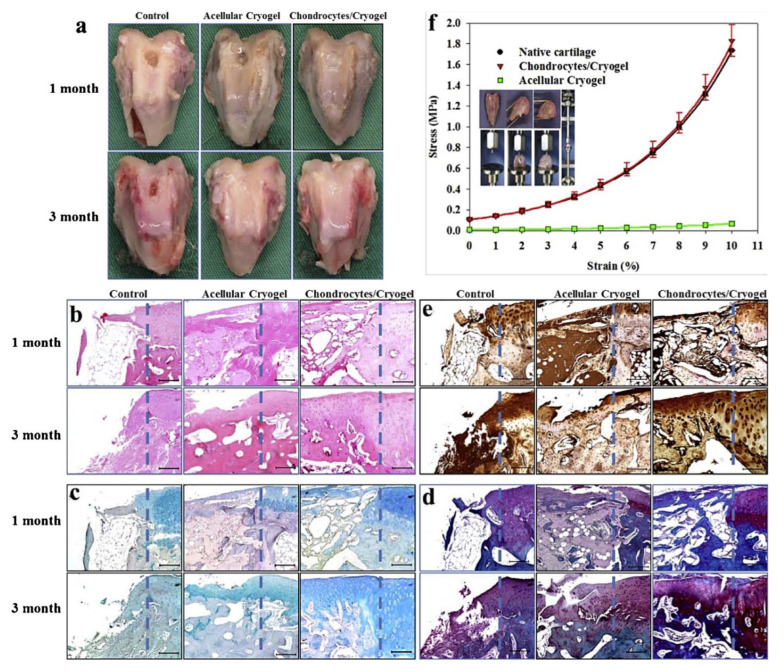
Gross observation (**a**), hematoxylin and eosin (H&E) (**b**), Alcian blue (**c**), Safranin O (**d**) and collagen type II immunohistochemical (**e**) staining of the explanted samples 1 and 3 months post-implantation. The rabbit cartilage defect was not treated (control), filled with gelatin/chondroitin-6-sulfate/hyaluronan/chitosan cryogel (acellular cryogel), or chondrocytes-seeded gelatin/chondroitin-6-sulfate/hyaluronan/chitosan cryogel (chondrocytes/cryogel). The defect creation boundary is shown as the dotted line in each panel with native cartilage to the right. Bar = 200 m. (**f**) Comparison of the stress–strain curves of native cartilage, acellular cryogel and chondrocytes/cryogel 3 months post-implantation. The lines are best-fit curves from Equation (1). The insert illustrates the setup for mechanical testing (reprinted with the permission from [83]).

He and co-workers [82] formed chondrocyte-laden, elastic, highly interconnected macroporous cryogel networks of HA and glycidyl methacrylate preserving cell viability and metabolic activity following cryogel administration via a syringe needle. Following 15 days of culture in cryogels, the chondrocytes exhibited enhanced ECM glysosaminoglycans (GAGs) in comparison with cells seeded in HA-based hydrogels. Furthermore, the production of collagen type II (COL II) indicated that the cells maintained their phenotype. The above demonstrated the potential of the injectable chondrocyte-laden cryogels to promote cartilage tissue regeneration. In another study, Shariatzadeh and co-workers [81] applied cryogelation for the fabrication of highly porous interpenetrating networks (IPNs) based on gelatin methacrylate (GelMA) (3 wt%) and HA (5–20 wt%). The developed cryogels were found to be mechanically stable to applied compression (up to 90%) and to exhibit shape-memory properties. Moreover, they were characterized by good cell adhesion and >90% cell viability, which entitled them to be promising cell carriers for soft tissue engineering (TE) applications. Fan and co-workers [85] combined cell cryopreservation with cryogelation for the development of cell-laden cryogels (CECG) exhibiting increased permeability and living space for cell growth, based on methacrylated HA. Both human mesenchymal stem cells (hMSCs) and chondrocytes were shown to be uniformly encapsulated within the CECGs and to retain their viability. Cryogels were revealed to promote chondrocyte proliferation and the secretion of ECM as well as the spreading and proliferation of hMSCs in comparison with HA-based hydrogels. Similarly, the culture of rabbit chondrocytes on MeHA-based cryogels resulted in enhanced collagen type II gene expression and the accumulation of collagen [84]. Kuo and co-workers [83] formed chondrocyte-laden cryogels based on gelatin–chondroitin sulfate–HA (GCH) and chitosan–gelatin–chondroitin sulfate–HA (GCH–chitosan) for the repair of cartilage defects. Chondrocytes were shown to proliferate/differentiate in both cryogels. In the case of GCH–chitosan cryogel, chitosan appeared to reduce cell proliferation and to upregulate secretion of GAGs and COL II. Cryogel implantation in a full-thickness cartilage lesion was revealed to effectively regenerate tissue (Figure 8). Likewise, the incorporation of glucosamine (GlcN) (9 or 16 wt%) in gelatin–HA (GH) cryogels was shown to influence cell proliferation and morphology and to aid in preserving the chondrogenic phenotype in vitro. The implantation of chondrocyte-laden GH–GlcN cryogels into full-thickness cartilage lesions in rabbits was found to induce the formation of neocartilage tissue with positive staining for COL II and glycosaminoglycans (GAGs) [86].

#### 4.1.3. Microgels

**Figure 9:**
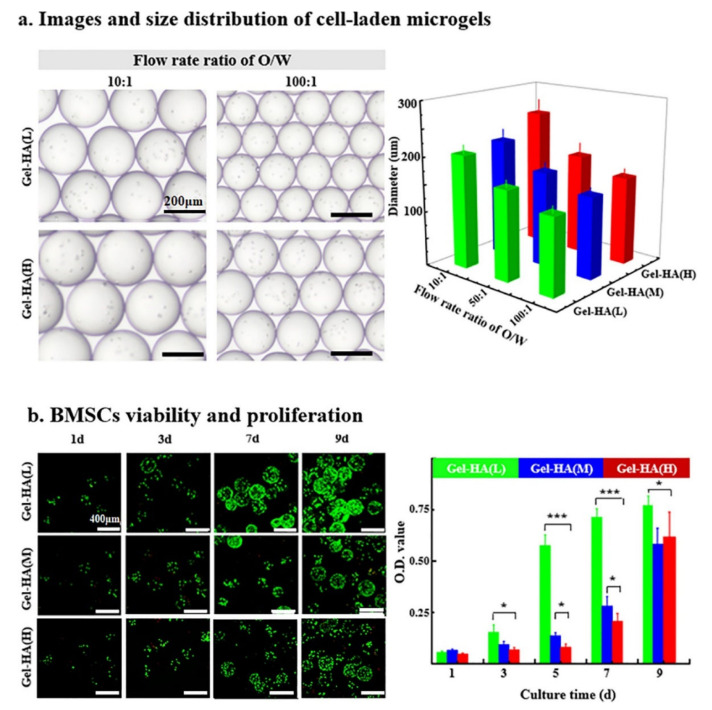
Microfluidic fabrication and characterization of BMSC-laden gel-HA microgels: (**a**) effects of flow rate ratios of oil/water on the diameter of BMSC-laden gel-HA microgels; (**b**) BMSC viability and proliferation behaviors in gel-HA microgels (reprinted with the permission from [87]).

In spite of the capability of hydrogels to mimic the extracellular matrix (ECM), their large size (i.e., low surface to volume ratio resulting in small diffusion area and long diffusion distance for soluble molecules) could hinder the uniform distribution of biophysical cues/nutrients leading to a biochemical gradient within the microgels and thus impeding high-throughput screening and evaluation. In this respect, Feng and co-workers [87] proposed a microgel model (i.e., gelatin/HA microgels formed in microfluidic devices and exhibiting a low, medium and high degree of cross-linking) (Figure 9) mimicking the ECM microenvironment to examine in vitro the effect of mechanical cues embedded in the cellular microenvironment on the fate of bone marrow derived MSCs (BMSCs). BMSCs cultured in hydrogel beads of low cross-linking density were shown to differentiate to hyaline cartilage as opposed to those cultured in microgels with medium and high cross-linking density, which differentiated towards fibrocartilage, thus verifying the effect of the mechanical microenvironment on the proliferation, distribution and differentiation of MSCs.

Akkiraju and co-workers [88] prepared HA-based hydrogel particles (HGPs) encapsulating a newly designed bone morphogenetic protein receptor type I (BMPRI) mimetic peptide, namely CK2.1. The intra-articular injection of pluripotent MSCs-laden HA-CK2.1 hydrogel particles in mice with osteoarthritis (OA)-like articular cartilage damage resulted in the total repair of the cartilage defects without induction of hypertrophy in contrast to cell-laden blank HGPs, which resulted in the enhanced production of collagen type X and osteocalcin. This could be attributed to the sustained release of the BMPRI mimetic peptide. In another study, HA-based hydrogel particles were decorated with heparin (HP) without affecting its ability to bind to bone morphogenetic protein 2 (BMP-2). BMP-2 release from the hybrid particles (HA/HP) was shown to follow near zero-order kinetics. In vitro culture of murine MSCs in the presence of BMP-2 loaded HA/HP hydrogel particles resulted in the upregulation of chondrogenic markers and production of cartilage-specific ECM components [89].

### 4.2. Three-Dimensional Bioprinted Hydrogel Constructs

**Figure 10:**
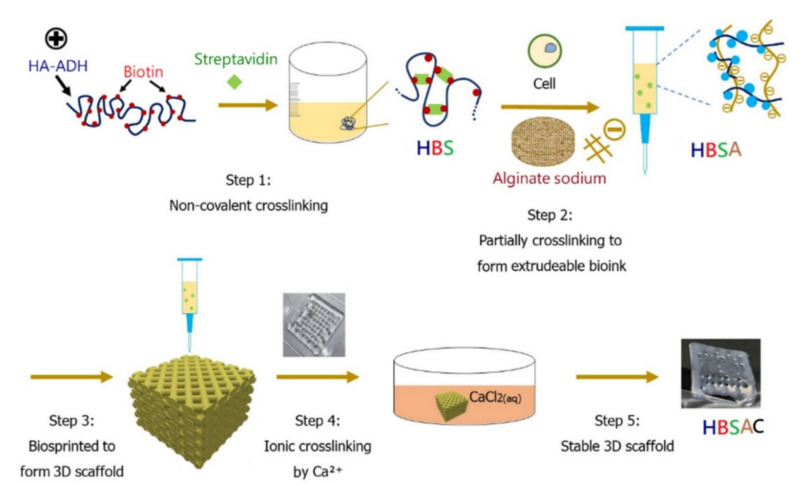
The strategy of 3D bioprinting with double cross-linking steps was as follows: the first cross-linking step involved the partial cross-linking of HA–ADH–biotin–streptavidin (HBS) hydrogel, and the HBS mixed with sodium alginate served as a hybrid bioink (HBSA) for the second ionic cross-linking step with Ca2+ ions. The 3D HBSA scaffolds after printing were submerged in CaCl2 solution to achieve ionic cross-linking to form an HBSAC hydrogel construct through ion transfer (reprinted with the permission from [99]).

Three-dimensional bioprinting (3DBP) is a scalable additive manufacturing technique applied to regenerative medicine for the spatially controlled layer-by-layer fabrication of cell-laden scaffolds. It combines 3D printing features such as the controlled design, fabrication and modelling of the constructed scaffold, with the precise patterning of living cells within the construct. Due to its internal/external spatial arrangement, the engineered construct can be integrated with native tissues, mature into equivalents of functional tissues (e.g., osteochondral tissue) and repair lesions of various sizes, thicknesses and geometries. In addition, it can be used for the construction of organ analogs [90,91,92,93,94,95,96]. Finally, 3DBP techniques (e.g., inkject, laser-induced forward transfer, extrusion-based bioprinting) are suitable for implementing spatial variations such as the zonal structure of the articular cartilage in engineered constructs [92,97]. However, it should be noted that the selection of the most suitable bioinks for 3DBP is still challenging [97]. Bioinks are materials mimicking the extracellular matrix (ECM) environment and promoting cell adhesion, proliferation and differentiation [95]. Presently, the selection of bioinks is mainly dependent on their biocompatibility with cells (i.e., cell viability, promotion of cell growth, proliferation and/or differentiation) and their printing characteristics (e.g., rheological properties, extrudability, stability after printing) [98]. They can be in the form of hydrogels (e.g., HA, alginate, gelatin), cell pellets, tissue strands or spheroids and decellularized ECM [91,93,95]. Hydrogels are considered promising materials for bioinks due to their high water content providing sustenance and facilitating the entrapment of cells and biological cues such as growth factors and proteins [97]. However, bioprinting cell-laden hydrogels exhibiting the required properties (e.g., structural integrity, storage and compression moduli, cell compatibility, cell adhesion and chondrogenic differentiation) is still a significant issue affecting the application of 3DBP in cartilage tissue engineering (TE) [99]. The fine-tuning of bioink composition/mechanical properties and material processing parameters is critical for developing viable, biomechanically relevant cartilage substitutes [92,97]. In this respect, bioinks with increased polymer content are commonly employed to enable the fabrication of constructs with superior stability and anatomical accuracy [92,93,100]. On the other hand, this feature often impedes cell bioactivity and the homogeneous distribution of the produced ECM [93]. Antich and co-workers [92] developed a biomimetic hybrid scaffold for the repair of articular cartilage by coprinting a bioink consisting of hyaluronic acid (HA) and alginate with polylactic acid (PLA). The bioink was shown to be promising for 3DBP-based cartilage tissue engineering since it promoted chondrogenesis and increased the deposition of a specific matrix, thus resulting in enhanced neocartilage tissue formation. In another study, hydrogels of low polymer content (e.g., 3 wt%) comprising various compositions of thiolated HA and allyl-modified poly(glycidol) and incorporating human mesenchymal stromal cells were supplemented with 1 wt % HA of high molecular weight (MW) in order to be applied as bioinks in poly(ε-caprolactone) (PCL)-supported 3DBP. The presence of the high MW HA resulted in increased construct quality and improved the homogeneous distribution of ECM, independently of the 3DBP process, in contrast with high-polymer-content bioinks (e.g., 10 wt%), which resulted in pericellular ECM deposition [93]. To capture the fibrillar nature of ECM, its components need to be arranged at the microscopic or molecular level in contrast to the macroscopic level achieved by extrusion-based bioprinting [94]. In this respect, Schwab and co-workers [99] developed a bioink consisting of collagen type I (COL I) fibers uniformly distributed within a tyramine-modified HA viscoelastic matrix. The shear stress during bioprinting controlled the orientation of the COL I fibers in the construct, thus affecting the cell behavior [94]. Nedunchezian and co-workers combined two cross-linking steps with 3DBP in order to form an adipose-derived stem cells (ADSCs)-laden hybrid HA-based construct for cartilage repair (Figure 10). The developed construct was revealed to have a satisfactory biocompatibility profile and to exhibit enhanced chondroinductive properties in comparison with an HA hydrogel.

Stichler and co-workers [101] applied a double-printing methodology to develop robust scaffolds via polycaprolactone (PCL)-assisted 3DBP using as a bioink a hydrogel based on thiolene clickable poly(glycidol)s (PGs) and immobilized bioactive HAs in the presence of a high MW HA (1, 2.5 wt%). It was shown that bioprinting did not harm the embedded cells. Finally, robot-assisted in situ 3DBP using methacrylated HA enriched with acrylate-terminated four-arm polyethylene glycol as bioink for cartilage repair was reported by Ma and co-workers [102]. A six-degree-of-freedom (6-DOF) robot was employed. The printing accuracy was improved via the development of a fast tool center point (TCP) calibration method. In vivo tests with rabbits indicated the successful repair of osteochondral defects following in situ bioprinting. The regenerated cartilage tissue was shown to exhibit similar biochemical and biomechanical properties with those formed following implantation of the printed construct [102]. The evolution of this technology and the integration of high-resolution radiographic imaging and computer-aided design/manufacturing with real-time three-dimensional bioprinting could result in the future in the successful reconstruction of both articular cartilage and bone [91].

## 5. Clinical Evaluation

Regardless of the numerous hyaluronic acid (HA)-based hydrogels that have been developed for the repair of cartilage defects and have shown encouraging results in in vitro and in vivo tests, only one medicinal product (Cartistem^®^) comprising culture-expanded allogeneic human umbilical-cord-blood-derived mesenchymal stem cells (hUCB-MSCs) and an HA hydrogel has been evaluated in phase I/II [103,104] and III [105] clinical trials for its ability to regenerate cartilage tissue. More specifically, Cartistem^®^ has been assessed concerning its efficiency to restore large cartilage lesions caused by injury, ageing or degenerative diseases, and to achieve long term clinical improvement. The performance of Cartistem^®^ was also compared with microfracture, a method applied successfully for the repair of small cartilage defects and not so successfully for the repair of large cartilage lesions [105] (Table 3). Apart from Cartistem^®^, a limited number of acellular HA-based hydrogels (e.g., Hydros, Hydros-TA [106], Gel-One^®^ [107,108,109,110], HYA-JOINT Plus [111], hylastan SGL-80 [112], Cingal^®^ [113], Monovisc^®^ [113,114], Durolane^®^ [115,116,117], XLHA [118] and Synovian^®^ [119]) have also been evaluated in clinical trials regarding their (long-lasting) analgesic efficiency and improvement of physical function in comparison with typical HA-based viscosupplements and corticosteroids in patients with knee OA (Table 3).

## 6. Conclusions

Numerous hMSC- or chondrocyte-laden HA-based injectable hydrogels (e.g., in situ forming hydrogels, cryogels, microgels) and 3D-bioprinted hydrogel constructs of various compositions and functional groups, exhibiting variable rheological/mechanical properties and encapsulating or not bioactive molecules, have been developed and preclinically assessed during the last decade regarding their possible utilization in the regeneration of injured cartilage tissue. Most of the developed hydrogels were shown to enhance cell proliferation, maintain the chondrocyte phenotype and favor the chondrogenic differentiation of the encapsulated mesenchymal stem cells (MSCs) as well as promote the secretion of neocartilage extracellular matrix (ECM) in vitro. On the other hand, the in vivo assessment of HA-based injectable hydrogels and 3D-bioprinted hydrogel constructs revealed that mainly composite hydrogels/hydrogel constructs comprising HA among other components (e.g., methacrylated gelatin, chondroitin-6-sulfate, chitosan, hyperbranched polyethylene glycol diacrylate, thiolene clickable poly(glycidol)s, etc.) were efficient in repairing full thickness cartilage lesions in various animal models (e.g., rats, rabbits, etc.). The above-mentioned experimental observations indicate the need to combine HA with tissue adhesive materials and polymers of higher mechanical strength for CTE applications. However, at this point it should be noted that the complexity of the cartilage tissue, the biological and rheological/mechanical requirements for cell growth and proliferation, and the specific biological and/or physical (e.g., thickness) characteristics of the cartilage defects, hinder the formation of an optimal hydrogel. Finally, the lack of standardized in vivo assessment methods (e.g., variety of animal models, variations in cartilage defect thickness, hydrogel assessment in the absence of load bearing conditions, etc.) and experimental protocols impede the generation of comparable experimental data and the development of hydrogels for clinical applications. Accordingly, only a small number of HA-based hydrogels have reached the clinical development phase, with Cartistem^®^ being the only HA-based medicinal product that has been evaluated in phase I, II and III clinical trials regarding its ability to regenerate cartilage tissue.

## 7. Future Perspectives

The above-mentioned challenges need to be surpassed in order to allow cell-laden HA-based injectable hydrogels and 3D-bioprinted hydrogel constructs to play a key role in the treatment of cartilage defects. In this respect, novel biomaterial combinations should be selected for the development of hydrogels based on high-throughput systematic screening of numerous HA derivatives (existing and newly synthesized) and other natural or synthetic materials exhibiting synergetic properties. Furthermore, progress in the scientific areas of tissue engineering, cell culture, gene expression and smartly designed biomimetic hydrogels should be combined with better understanding of hydrogel properties, cartilage physiology and mechanisms governing neocartilage tissue formation as well as improved fabrication protocols and animal models better representing human pathology. The integration of high-throughput screening, predictive mathematical models and in vitro/in vivo assays should allow the selection of the optimum hydrogel characteristics, leading to a rational design of HA-based hydrogels for CTE applications. Finally, with respect to 3D bioprinting, the evolution of this technology and the integration of high-resolution radiographic imaging and computer-aided design/manufacturing with real-time in situ bioprinting could result in the future in the successful reconstruction of articular cartilage.

## Figures and Tables

**Table 1:** Advantages and disadvantages of natural materials used in cartilage tissue engineering [6,28,29,30,31,32].

Material	Water Solubility	Electrostatic Charge	Functional Group	Cross-Linking Type	Advantages	Disadvantages
*Polysaccharides*						
Hyaluronic Acid	Soluble	Negative	-COOH, -OH, -CH_3_CO	Ionic, chemical	Main component of cartilage tissue Easy modification, functionalization and/or combination with other biomaterials Interaction with the cell surface receptors CD44 ^a^, RHAMM ^b^ and ICAM-1 ^c^ Supports the growth of chondrocytes Promotes the differentiation of MSCs ^d^ towards a chondrogenic phenotype Enhanced neocartilage tissue formation	Poor mechanical properties Rapid degradation by hyaluronidase Probability of inflammation after degradation of low-MW ^e^ HA ^f^ fragments
Chondroitin sulfate	Soluble	Negative	-COOH, -OH	Ionic, chemical	Component of cartilage Hypertrophy regulation during MSCs ^d^ chondrogenesis Promotes the deposition of cartilage ECM ^g^	Rapid degradation
Chitosan	Insoluble; soluble in acetic acid (pH < 4)	Positive at pH < 5.8	-NH_2_, -OH	Ionic, chemical	Low cost Antibacterial properties Structural similarities with GAG ^h^	Low cell–matrix interaction Ionic-cross-linked hydrogel of low stability
Alginate	Soluble	Negative	-COOH, -OH	Ionic, chemical	Low cost	Low cell–matrix interaction
Agarose	Soluble in hot water	Neutral	-OH	Temperature-dependent	Low cost	Reduced bioactivity
***Proteins***						
Collagen	Soluble	Neutral	-COOH, -NH_2_, -OH	Physical, ionic, chemical	Sufficient cell–matrix interaction	Possibility for antigenicity
Gelatin	Soluble	Neutral	-COOH, -NH_2_, -OH	Ionic, chemical	Presence in cartilage tissue Adequate cell–matrix interaction	Rapid enzymatic degradation
Silk fibroin	Soluble	Neutral	-COOH, -NH_2_	Sol–gel transition	Increased mechanical strength	Possibility for antigenicity Low biodegradability of β-sheet crystals
Fibrin	Soluble	Neutral		Assembly of polypeptides into fibrin via thrombin-mediated cleavage of fibrinogen	Absence of toxic cross-linkers	Rapid enzymatic degradation

^a^ Cluster determinant 44, ^b^ hyaluronic-acid-mediated motion receptor, ^c^ intercellular adhesion molecule-1, ^d^ mesenchymal stem cells, ^e^ molecular weight, ^f^ hyaluronic acid, ^g^ extracellular matrix, ^h^ glycosaminoglycan.

**Table 2:** Preclinical evaluation of in situ forming injectable HA-based hydrogels.

Material	HA ^a^ MW ^b^ (KDa)/ Functional Groups	DM (%) ^c^/ Functionalization	Cross-Linking Reaction/Cross-Linker/Gelation Onset (s)	Bioactive Agent/Stimulation/Extra	Cell Type/Cell Number per mL	Outcome
*Redox/enzymatic reaction*						
HTG ^d^ [57]	-/COOH	13.38/-	Enzymatic/tyrosinase/108–132	EGCG ^e^/-	Porcine chondrocytes/2 × 10^7^	The HTG ^d^ hydrogel was found to promote accumulation of ECM ^f^ EGCG ^e^-loaded hydrogel protected cartilage from inflammation and diminished cartilage loss in an OA ^g^ mouse model
HA-GEL ^h^ [59]	350/COOH	-/-	Redox/HRP ^i^ and H_2_O_2_/-	-/electrical	Porcine MSCs ^j^/1 × 10^6^	The electrical stimulation was revealed to enhance the chondrogenic potential of the HA-GEL ^h^ hydrogel
HA-TA ^k^ [52]	70/COOH	24	Oxidative coupling reaction (redox)/HRP ^i^ and H_2_O_2_/10–500	Platelet lysate	MSCs ^j^/10^7^	This study showed that hMSC-laden HA-TA ^k^ hydrogels that were enriched with platelet lysate favored a cartilage-like ECM ^f^ deposition in vitro. Hydrogel degradation happened at the same time with ECM ^f^ deposition leading to the formation of a dense matrix. The results of this study confirmed the possibility of using HA-TA-PL ^l^ hydrogel as a cell delivery system for cartilage tissue engineering applications.
HA ^a^ [60]	1010–1800/COOH	/transglutaminase substrate peptides	Enzymatic/thrombin, factor XIII, transglutaminase-modified heparin/60–120	TGF-β ^m^/-	Human infant chondrocytes/5, 10 or 15 × 10^6^,	Cartilaginous matrix was produced by polydactyly chondrocytes in the developed biomimetic hydrogels
HA ^a^ [50]	1010–1800/COOH	/transglutaminase substrate peptides, heparin	Enzymatic/thrombin, transglutaminase factor XIII/900	TGF-β ^m^/-	Human chondroprogenitor cells (fetal origin)/15 × 10^6^	Matrix deposition was shown to be stimulated by a slow release of TGF-β ^m^.
HA-MA-PNIPAAm-CL ^n^ [61]	2000/OH	30/-	Redox/-/-	-/-	Rabbit adipose-derived stem cells/1 × 10^6^	Enhancement of chondrogenesis of adipose-derived stem cells in HA-PNIPAAm-CL ^n^ hydrogel for cartilage regeneration in rabbits
PVCL-g-HA ^o^ (methacrylate HA) [62]	58 and 1100/OH	-/-	Redox/VA-057 ^p^ initiator/-	-/-	Bovine chondrocytes/3.65 × 10^6^	In this study, thermosensitive injectable hydrogels were developed to be used for cartilage tissue engineering applications. These hydrogels appeared to be promising materials favoring the viability of chondrocytes as well as the biochemical synthesis of ECM ^f^ proteins under hypoxia.
HA-Tyr ^k^ [63]	90/COOH	6/-	Oxidative coupling reaction (Redox)/HRP ^i^ and H_2_O_2_/60		Caprine MSCs ^j^/10^7^	The 3D microenvironment of the HA-Tyr ^k^ hydrogels controlled cellular condensation throughout chondrogenesis and influenced the spatial organization of cells, ECM ^f^ biosynthesis and histogenesis of cartilage tissue.
***Michael-type addition reaction***						
MeHA ^q^ [2]	66–99/OH	46.5 ± 5.5/- 46.5 ± 5.5/CS ^r^-binding peptide 46.5 ± 5.5/-	Michael-type addition/MMP7 ^s^-degradable peptide/457 ± 68.1	-/-	MSCs ^j^/1 × 10^6^ chondrocytes/1.25 × 10^6^	Differentiation of MSCs ^j^ towards a chondrogenic phenotype Enhancement of cell differentiation towards a chondrogenic phenotype Arrangement of cell clusters in isogenous groups, distinctive of hyaline cartilage morphology and deposition of glycosaminoglycans
Hyper-branched PEGDA ^t^-thiolated HA [56]	-/COOH	-/-	Michael-type addition/-/120	-/-	Human AFF-MSCs ^u^/5 × 10^6^	AFF-MSCs ^u^ were differentiated towards a chondrogenic phenotype Full-thickness cartilage defects were successfully repaired in 8 weeks.
MA-HA ^v^ [64]	59/COOH	30/-	Michael-type addition/MMP ^w^-cleavable peptides/-	-/-	Human MSCs ^j^/20 × 10^6^	Enhanced chondrogenesis and suppressed hypertrophy of human MSCs ^j^ encapsulated in MA-HA ^v^ hydrogels were the result of cell-mediated hydrogel degradation via MMPs ^w^.
***Schiff base reaction***						
Glycol chitosan-oxidized HA ^a^ [65]	100/OH	33.4/-	Schiff base reaction/-/-	Cartilage ECM ^f^ particles/-	BMSCs ^x^/2 × 10^7^	The presence of cartilage ECM ^f^ particles resulted in the formation of more mature cartilage tissue containing higher levels of GAGs ^y^ and collagen II
Collagen-HAD ^z^ [66]	1500–1800/	-/-	Schiff base reaction/-/-	-/-	Rabbit chondrocytes/5 × 10^4^	Both healthy and osteoarthritic cartilage in vitro models were developed by varying HAD ^z^ concentration in the hydrogels.
CH-HAD ^aa^ [53]	-/OH	50/-	Schiff base raction//25–60	-/-	Rabbit chondrocytes/5 × 10^6^	The results of this study demonstrated that hydrogel stiffness had a huge impact on maintaining the phenotype of chondrocytes as well as the production of ECM ^f^.
OHA/GC ^ab^ [67]	1000/OH	~6.8–33.8/-	Schiff base raction//97–120	-/-	ATDC5 chondrogenic cell line/10^6^	OHA/GC ^ab^ hydrogels exhibited efficient biocompatibility and resistance under natural conditions, and they could be used as an injectable cell delivery system for cartilage tissue engineering applications.
***Photocross-linking***						
AHAMA ^ac^ [55]	100–200/OH	24/-	Photopolymerization/Irgacure 2959/-	-/-	BMSCs ^x^/5 × 10^6^	AHAMA ^ac^ hydrogel was shown to significantly promote neocartilage integration with host tissue and cartilage regeneration in osteochondral defects in rats.
mGL/mHA ^ad^ [58]	66–99/OH		Photocross-linking/LAP ^ae^		Human BMSCs ^x^/ 20 × 10^6^	Chondrogenesis and cartilage formation were favored for MSCs ^j^ encapsulated in mGL/mHA ^ad^ hydrogels at a ratio of 9:1. The implantation of mGL/mHA ^ad^ hydrogel inside the defect exhibited cartilage and bone formation after 12 weeks, indicating its potential use for the repair of osteochondral defects.
GelMA ^af^/HAMA ^ag^ [68]	860/OH		Photocross-linking/LAP ^ae^ and visible light (405 nm), Irgacure 2959 and UV ^ah^ light (365 nm)	/MEW-mPCL ^ai^ reinforcement	Human articular chondrocytes/10^7^	In this study, photocross-linking based on UV ^ah^ light exhibited enhanced chondrocyte cell behavior compared to visible light cross-linking. Bovine-derived GelMA ^af^ photocross-linked with Irgacure 2959 showed properties that resembled native articular cartilage tissue.
MeHA ^ag^ [69]	75/OH	37/± HAV, ADAM-cleavable domain	Photocross-linking/Irgacure 2959		MSCs ^j^/20 × 10^6^	This study showed the possibility of a hydrogel material mimicking the complicated microenvironment throughout embryogenesis towards the formation of stem-cell-based cartilage.
MeHA ^ag^/ELP ^aj^ [70]	1600/OH		Photocross-linking/	ZnO ^ak^ (antimicrobial)	Human MSCs ^j^, NIH-3T3/2 × 10^6^, 5 × 10^6^	This study confirmed that MeHA ^ag^/ELP ^aj^-ZnO ^ak^ hydrogels can be used for tissue engineering applications due to their tunable natural characteristics and their adhesive and antimicrobial properties.
MeHA ^ag^ [71]	1000/OH	1.2/	Photocross-linking/Irgacure 2959	TGFβ3 ^m^/DCC ^al^ or DVC ^am^ microparticles	Rat BMSC ^x^/20 × 10^6^	This study demonstrated that DVC ^am^ microparticles showed enhanced chondroinductivity and rheological performance of hydrogel precursors in comparison to DCC ^al^.
MeHA ^ag^ [72]	74/OH		Photocross-linking/Irgacure 2959	TGFβ3 ^m^	Allogeneic MSCs ^j^/60 × 10^6^	The data from this study indicated that combining MSCs ^j^ with growth factors and hydrogel materials followed by a preculture period and utilizing standard tissue engineering techniques could give a more promising outcome in comparison with directly implanting cells and growth factors.
MeHA ^ag^, MeHA ^ag^+ColI ^an^, MeHA ^ag^+MeCS ^ao^ [73]	74/OH	30	Photocross-linking/Irgacure 2959		Human MSCs ^j^/20 × 10^6^	The results of this study showed that by controlling the formula of cartilage specific biopolymers embedded into cell-laden hydrogels, it was possible to tune the level of maturation and calcification of the newly formed cartilage.
MeHA ^ag^ [74]	74/OH	29	Photocross-linking/Irgacure 2959		Human MSCs ^j^/20 × 10^6^	The study showed that HA ^a^ concentration, and not cross-linking density, can affect the hypoxia-mediated positive or negative control of the hypertrophic differentiation of cells encapsulated in HA ^a^ hydrogels after chondrogenic induction. The outcome of this study could be useful for the design and optimization of hydrogels and tissue culture protocols.
Fibrinogen/HA-MA ^ag^ [75]	1500–1800/OH	95 ± 13/-	Ionic and chemical interactions, Photocross-linking/Irgacure 2959/	TGFβ ^m^/-	BMSCs ^x^/10^4^/well	Fibrin/HA-MA ^ag^ hydrogel could be used for the delivery of BMSCs ^x^. Fibrin/HA-MA ^ag^ hydrogel favors the differentiation of BMSC ^x^ into chondrocytes and it could be helpful for the repair of articular cartilage tissue in OA ^g^ patients.
GelMA ^af^ and HA-MA ^ag^ [76]	860/OH		Photocross-linking/Irgacure 2959/900		Human chondrocytes/10^7^	The mixtures of GelMA ^af^ and HA-MA ^ag^ are considered promising materials for cartilage tissue engineering applications.
MeHA ^ag^ [77]	74/OH	27	Photocross-linking/Irgacure 2959		MSCs ^j^ and/or chondrocytes/20 × 10^6^	The study demonstrated that the coculture of hMSCs ^j^ and chondrocytes encapsulated in HA ^a^ hydrogels increased the mechanical properties and cartilage-specific ECM ^f^ content of tissue-engineered cartilage.
***Self-cross-linking and other reactions***						
ColI ^an^/HA-sNHS ^ap^ [78]	61/COOH	32, 50, 83/	Self-cross-linking/no initiators and no cross-linkers/93–130		Rabbit chondrocytes/5 × 10^6^	These self-cross-linkable and injectable hydrogels with adjustable physical properties could be used for cartilage tissue engineering applications.
HA-SH ^aq^/GelSH ^ar^, HA-SH ^aq^/GelMA ^af^, HA-SH ^aq^/Gel ^as^ [79]	340/COOH	35.3/	Strong disulfide bonding between HA-SH ^aq^ and GelSH ^ar^/7.19, Michael addition between HA-SH ^aq^ and GelMA ^af^/7.31, Physical interaction/7.27	-/-	Rabbit chondrocytes/3 × 10^6^	The strong disulfide bonding was shown to enhance the performance/biological function of the encapsulated chondrocytes
Thiolated HA ^a^—collagen [32]	100, 300, 1000/COOH	-/-	Formation of disulfide bonds/thiolated icariin/1800	-/-	Chondrocytes/5 × 10^6^	The developed hydrogels were found to facilitate cell proliferation, maintain the chondrocyte phenotype and promote the secretion of cartilage ECM ^j^.
Thiolated HA—collagen I [54]	300/COOH	-/-	Self-cross-linking/10	-/-	Rabbit chondrocytes/5 × 10^6^	The hydrogels facilitated cell adhesion and proliferation The encapsulated chondrocytes were shown to maintain their phenotype and to secrete a great amount of ECM ^j^.
HA ^a^-ADH ^at^/PAD ^au^, HA ^a^-ADH ^at^/PAD-RGD ^av^ [80]	740/COOH	41.5/-	Hydrazone reaction/PAD-RGD ^av^/112–399	-/-	Chondrocytes/6 × 10^6^	HA ^a^-ADH ^at^/PAD-RGD ^av^ hydrogel with a 5/5 weight ratio was characterized as the most promising microenvironment that could mimic host tissue and maintain chondrocyte phenotype as well as favoring chondrogenesis

^a^ Hyaluronic acid, ^b^ molecular weight, ^c^ degree of modification, ^d^ tyramine-modified hyaluronic acid-gelatin, ^e^ epigallocatechin-3-gallate, ^f^ extracellular matrix, ^g^ osteoarthritis, ^h^ tyramine-modified hyaluronic acid—tyramine-modified gelatin, ^i^ horseradish peroxidase, ^j^ mesenchymal stem cells, ^k^ hyaluronic acid tyramine hydrogel, ^l^ hyaluronic acid tyramine hydrogel with platelet lysate, ^m^ transforming growth factor beta, ^n^ methacrylated hyaluronic acid cross-linked poly(*N*-isopropylacrylamide), ^o^ poly(*N*-vinylcaprolactam) and methacrylated hyaluronic acid, ^p^ 2,20-azobis[*N*-(2-carboxyethyl)22-methylpropionamidine]hydrate, ^q^ methacrylated hyaluronic acid, ^r^ chondroitin sulfate, ^s^ matrix metalloproteinase 7, ^t^ poly(ethylene glycol) diacrylate, ^u^ arthroscopic flushing-fluid-derived mesenchymal stem cells, ^v^ maleimide-modified HA, ^w^ matrix metalloproteinase, ^x^ bone marrow mesenchymal stem cells, ^y^ glycosaminoglycans, ^z^ hyaluronic acid dialdehyde, ^aa^ chitosan–hyaluronic acid dialdehyde, ^ab^ oxidized hyaluronate/glycol chitosan, ^ac^ methacrylated aldehyde-modified hyaluronic acid, ^ad^ methacrylated gelatin-methacrylated hyaluronic acid, ^ae^ lithium phenyl-2,4,6-trimethylbenzoylphosphinate, ^af^ gelatin methacryloyl, ^ag^ hyaluronic acid methacrylate (or methacrylated hyaluronic acid), ^ah^ ultraviolet, ^ai^ melt-electrowritten medical-grade polycaprolactone, ^aj^ elastin-like polypeptide, ^ak^ zinc oxide, ^al^ decellularized cartilage, ^am^ devitalized cartilage, ^an^ type I collagen, ^ao^ methacrylated chondroitin sulfate, ^ap^ *N*-hydroxysulfosuccinimide-activated hyaluronic acid, ^aq^ thiolated hyaluronic acid, ^ar^ thiolated gelatin, ^as^ gelatin, ^at^ adipic dihydrazide, ^au^ pectin dialdehyde, ^av^ aldehyde groups of G4RGDS-grafted aldehyde pectin.

**Table 3:** Clinical evaluation of HA-based hydrogels.

Objective	Trial/Phase	Number/Age/BMI ^a^ (kg/m^2^)/K-L ^b^ Grade/WOMAC ^c^ (Pain)/Sex of Participants	Treatment	Administration Route/Dose/Clinical Evaluation	Results
To assess Hydros ^d^ and Hydros ^d^-TA ^e^ regarding their safety and initial performance in comparison with Synvisc-One ^f^ in patients with knee OA ^g^ [106]	Prospective, multicenter, randomized, double-blind feasibility trial/II	98/60 years (average)/29.0 (average)/II and III/50–90 mm (using VAS 0–100 mm)/male and female	Hydros ^d^ Hydros ^d^-TA ^e^ Synvisc-One	i.a. ^h^ injection/6 mL of Hydros ^d^ or Hydros ^d^-TA ^e^, or Synvisc-One ^f^, single dose/2, 6, 13 and 26 weeks p.i. ^i^	Well-tolerated injections. Reduced WOMAC ^c^ A (pain) score in 26 weeks with all three formulations. Quicker pain relief with Hydros ^d^-TA ^e^ in comparison with Hydros ^d^ Enhancement in pain relief with Hydros ^d^-TA ^e^ in comparison with Synvisc-One ^f^
To investigate the safety and efficiency of Gel-One^® j^ in treating patients with symptomatic knee OA ^g^ [107] To examine the continued safety and efficacy of Gel-One^® j^(extension of the aforementioned clinical trial) [108]	Double-blind, multicenter, RCT ^k^/-Multicenter extension and retreatment trial	Gel-One^® j^: 247, PBS ^l^: 128/40–80 years old/28.3/I, II and III/≥40 mm (using VAS ^m^ 0–100 mm)/male and femaleContinued observation/≥ 64, second injection/≥ 196/40–80 years old/28.8/I, II and III/≥40 mm (using VAS ^m^ 0–100 mm)/male and female	Gel-One^® j^ PBS ^l^ (control) Gel-One^® j^ PBS ^l^ (control)	i.a. ^h^ injection/3 mL (30 mg HA ^n^/3 mL), 3 mL PBS ^l^, single dose/1 wk, 3, 6, 9 and 13 wks p.i. ^i^Second injection: i.a. ^h^ injection/3 mL (30 mg cross-linked HA ^n^/3 mL), 3 mL PBS ^l^, single dose/13 wks p.i. ^i^	Significant clinical improvement with respect to pain as well as physical function as early as 3 weeks Well-tolerated treatment Pain relief over 13 weeks Continued observation: improved OA ^g^ signs/symptoms over 26 weeks Well-tolerated and safe retreatment Retreatment efficiency similar to that of initial treatment for a time period of 13 weeks The initial injection was adequately effective to eliminate the need for a second injection in a large number of patients [120]
Integrated analysis of two RCTs ^k^ aiming to investigate the safety and efficiency of a single i.a. ^h^ injection of Gel-One^® j^ in treating knee OA ^g^ [109]	Multicenter, double-blind RCT ^k^/- Multicenter, double-blind RCT ^k^/-	SI-6606/01: -/60 years old (average)/~28.8 (average)/I-III/≥40 mm (using VAS ^m^ 0–100 mm)/male and female Gel/1133: -/60 years old (average)/~28.8 (average)/I-III/≥40 mm (using VAS ^m^ 0–100 mm)/male and female Pooled ITT ^o^ population: Gel-One^® j^: 649, PBS ^l^: 5345	Gel-One^® j^ PBS ^l^ (control) Gel-One^® j^ PBS ^l^ (control)	i.a. ^h^ injection/single dose/3, 6, 9 and 13 wks p.i. ^i^i.a. ^h^ injection/single dose/3, 6, 12, 18 and 26 wks p.i. ^i^	Proof of the efficiency of a single i.a. ^h^ injection of Gel-One^® j^ for the treatment of knee OA ^g^ over 26 weeks No major safety issues
To demonstrate the benefit of a single i.a. injection of Gel-One^® j^ as treatment of knee OA ^g^ in a population similar to those of viscosupplementation-reported trials [110]	Subgroup analysis of a multicenter RCT ^k^	Subgroup: 311 (Gel-One^® j^:152, PBS:159)/40–80 years old/II and III/40–80 mm (using VAS ^m^ 0–100 mm)/male and female	Gel-One^® j^ PBS ^l^ (control)	i.a. ^h^ injection/single dose/3, 6, 12, 18 and 26 wks p.i. ^i^	Clinically important pain improvement 26 weeks p.i. ^i^
To compare the safety and efficiency of HYA-JOINT Plus ^p^ with Synvisc-One ^f^ in subjects with kneeOA ^g^ [111]	Prospective, double-blind RCT ^k^/-	HYA-JOINT Plus ^p^: 62, Synvisc-One ^f^: 59/40–85 years old/~25 (average)/II, III//≥30 mm (using VAS ^m^ 0–100 mm)/male and female	HYA-JOINT Plus ^p^ Synvisc-One ^f^	i.a. ^h^ injection/3 mL of HYA-JOINT Plus ^p^ (20 mg/mL), 6 mL of Synvisc-One ^f^ (8 mg/mL), single dose/1, 3 and 6 months p.i. ^i^	Safe and efficient treatment for the time frame tested Significantly improved pain score compared with Synvisc-One ^f^ at 1, 3, and 6 months p.i. ^i^
To examine the efficacy of hylastan SGL-80 ^q^ regarding pain reduction in patients with knee OA ^g^, in comparison with corticosteroid injection [112]	Multicenter, double-blind, randomized, parallel group, trial/-	Hylasatan SGL-80 ^q^ (single dose): 130, hylasatan SGL-80 ^q^ (double dose): 129, methylprednisolone acetate: 132/>40 years old/-/I–III/1.5–3.5 (using Likert scale)/male and female	hylastan SGL-80 ^q^methylprednisolone acetate	i.a. ^h^ injection/4 mL of hylastan SGL-80 ^q^ on day 0, or 2 × 4 mL of hylastan SGL-80 ^q^ on day 0 and week 2, or 40 mg of methylprednisolone acetate on Day 0/4, 8, 12, 16, 20 and 26 weeks	Significantly reduced pain with all three treatments No safety issues Hylastan SGL-80 ^q^ was not proven to be superior to methylprednisolone acetate
To evaluate the efficacy and safety of Cingal^® r^ in comparison with Monovisc^® s^ for the treatment of knee OA ^g^ [113]	Prospective, randomized, multicenter, double-blind, placebo-controlled trial/-	Cingal^® r^:149, Monovisc^® s^:150, saline:69/40–75 years old/40–90/I, II or III/40–90 mm (using VAS ^m^ 0–100)/male and female	Cingal^® r^ Monovisc^® s^ Saline	i.a. ^c^ injection/4 mL of Cingal^® r^ (88 mg cross-linked HA and 18 mg TH), 4 mL of Monovisc^® s^ (88 mg cross-linked HA), 4 mL of saline, single dose/1, 3, 6, 12, 18 and 26 wks p.i. ^i^	Significantly better performance of Cingal^® r^ compared with Monovisc^® s^ from 1 to 3 weeks Similar performance of Cingal^® r^ and Monovisc^® s^ from 6 to 26 weeks.
To prove the safety and efficacy of Monovisc^® s^ in relieving joint pain inidiopathic knee OA ^g^ patients [114]	Multicenter, double-blind, randomized, placebo-controlled trial/-	Monovisc^® s^: 184, saline: 185/35–75 years old/20–40 kg/m^2^/II or III/200–400 mm (VAS ^m^ pain score 0–500 mm)/male and female	Monovisc^® s^ Saline	i.a. ^h^ injection/4 mL of Monovisc^® s^, 4 mL of saline (0.9%), single dose/2, 4, 8, 12, 20 and 26 wks p.i. ^i^	Safe and efficient treatment Clinically meaningful pain reduction in 2 weeks ≥50% improvement in WOMAC ^c^ pain by week 26
To assess thesafety and efficiency of Durolane^® t^ in unilateral knee OA ^g^ patients [115]	Randomized, double-blind, saline-controlled trial/-	Durolane^® t^: 108, saline: 110/> 50 years old/20.1–41/Likert version of WOMAC ^c^ pain score: 7–17/male and female	Durolane^® t^ Saline	i.a. ^c^ injection/3 mL of Durolane^® t^ (20 mg/mL) or 3 mL saline, single dose/2, 4 and 6 wks p.i. ^i^	Well-tolerated treatment No significant difference between Durolane^® t^ and control at 6 weeks (primary analysis) Significantly higher responder rate with Durolane^® t^ at 6 weeks compared with control for patients with no clinical effusion in the knee at baseline (post hoc subgroup analysis)
To compare Durolane^® t^ with MPA ^u^ for the treatment of unilateralknee OA ^g^ [116]	Prospective, multicenter, randomized, active-controlled, double-blind, noninferiority trial (blinded phase)Open label extension phase	Durolane^® t^: 221, MPA ^u^: 221/35–80 years old/≤40/II, III/7–17/male and female	Durolane^® t^ MPA ^u^	Blinded phase: i.a. ^h^ injection/3 mL of Durolane^® t^ (20 mg/mL) or 1 mL of MPA ^u^ (40 mg/mL), single dose/2, 4, 6, 12, 18 and 26 wks p.i. ^i^OLE ^v^: i.a. ^h^ injection/3 mL of Durolane^® t^ (20 mg/mL), single dose/28, 39 and 52 wks post initial i.a. ^h^	Well-tolerated treatment and noninferior compared with MPA ^u^ at 12 weeks Benefit maintenance up to 26 weeks in contrast to MPA ^u^ Second i.a. ^h^ injection at 26 weeks resulted in long-term improvement with no risk of complications or increased sensitivity
To compare safety and effectiveness of Durolane^® t^ and Artz ^w^ in treating knee OA ^g^ [117]	Multicenter, randomized, double-blind, noninferiority trial/-	Durolane ^® t^:175, Artz ^w^:174/40–80 years old/-/II or III/7–17 (Likert pain score range 0–20)/male and female	Durolane^® t^ Artz ^w^	i.a. ^h^ injection/1 × 3 mL of Durolane^® t^ (and 4 sham s.c. ^x^ injections on weeks 1, 2, 3 and 4); or 5 × 2.5 mL of Artz ^w^ on weeks 0, 1, 2, 3 and 4/0, 6, 10, 14, 18 and 26 wks	Safe, well-tolerated and efficient treatments A single dose of Durolane^® t^ is not inferior to multiple injections of Artz ^w^ regarding pain, stiffness, function and global self-assessment, at 18 and 26 weeks
To evaluate the safety and efficiency of XLHA ^y^ in comparison with HMWHA ^z^in treating symptomatic knee OA ^g^ [118]	Double-blind, randomized, multicenter, noninferiority trial	XLHA ^y^ (single dose): 141, HMWHA ^z^ (three doses): 146/>40 years old/<32/I-III/≥40 mm (using VAS ^m^ 0–100 mm)/male and female	XLHA ^y^ HMWHA ^z^	i.a. ^h^ injection/XLHA ^y^ group: 2 × 2 mL of PBS ^l^ (9 mg/mL) and 3 mL of XLHA ^y^ (20 mg/mL), HMWHA ^z^ group: 3 × 2 mL of HMWHA ^z^ (10 mg/mL)/1 wk, 2, 3, 4, 9, 12 and 15 wks p.i. ^i^	A single i.a. ^h^ injection of XLHA ^y^ was not found to be inferior to three weekly i.a. ^h^ injections of HMWHA ^z^ with respect to WBP ^aa^ reduction
To compare Conjuran^® ab^ with Synovian^® ac^and Hyruan Plus^® ad^ regarding their analgesic efficiency in patients with knee OA [119]	Pilot study	Synovian^® ac^: 5, Hyruan Plus^® ad^: 5, Conjuran^® ab^: 5/≥40 years old/-/I- III/≥40 mm (using VAS 0–100 mm)/male and female	Synovian^® ac^Hyruan Plus^® ad^Conjuran^® ab^	i.a. ^h^ injection/3 i.a. ^h^ injections at 1 week interval (all three groups), 3 mL of Synovian^® ac^ (20 mg/mL) and 2 × 3 mL of saline, 3 × 2 mL of Hyruan Plus^® ad^ (10 mg/mL), 3 × 2 mL of Conjuran^® ab^ (20 mg/mL)/4 wks after the last injection	Conjuran^® ab^ reduced more effectively WBP ^aa^ in comparison with Synovian^® ac^ and Hyruan Plus^® ad^
To examine the safety and efficiency of YYD302 ^ae^ for knee OA ^g^ [121]	Randomized, double-blind, active-controlled, multicenter trial/III	190/≥40 years old/≤32/I- III/≥40 mm (using VAS ^m^ 0–100 mm)/male and female	YYD302 ^ae^Synovian^® ac^	i.a. ^h^ injection/2 mL of YYD302 ^ae^, 3 mL of Synovian^® ac^, single dose/2, 4 and 12 wks after the i.a. ^h^ injection	
To examine the safety and efficiency of Cartistem^® af^ with respect to the regeneration of articular cartilage [103]	Open-label, single-arm, single-center trial/I/II	7/51–77 years old/-/III (ICRS ^ag^ grade of defect: 4)/40–60 mm (using VAS ^m^ 0–100 mm)/male and female	Cartistem^® af^	Transplantation, closure of wound and application of a splint/0.5 mL of Cartistem^® af^ per cm^2^ of defect (0.5 × 10^7^ cells per ml), low-dose: 2.3–2.5 mL of Cartistem^® af^, high dose: 3.3–4.0 mL of Cartistem^® af^/24 weeks (short term), 7 years (long term)	Efficient medicinal product for regeneration of robust cartilage The 7-year follow up revealed stable, improved clinical outcome and absence of significance adverse effects
To investigate the ability of Cartistem^® af^ to reliably restore cartilage in patients with large cartilage lesions and to examine the long-term maintenance of the potential clinical improvements [105]	Randomized controlled trial/III	Cartistem^® af^: 57, microfracture: 57/55.9 years old (average)/~ 26 (average)/II, III (ICRS ^ag^ grade 4)/-/male or female	Cartistem^® af^microfracture	Surgical implantation, closure of wound and application of a splint/-/48 weeks, 36, 48 and 60 months	Improvement of ICRS ^ag^ grade at 48 weeks No significant improvement of VAS ^m^ pain, IKDC ^ah^ and WOMAC ^c^ scores compared with microfracture at 48 weeks Significantly better clinical results in comparison with microfracture 36 and 60 months postsurgical intervention. Improved grade of cartilage in elderly patients with full-thickness cartilage lesions as well as improved cartilage function and pain 60 months post operation compared with microfracture
To evaluate the safety and efficiency of Cartistem^® af^, in treating articular cartilage lesions in the knee due to trauma, ageing, or degenerative diseases [104]	Open label trial/I/IIa	12/>18 years old/≤35/ICRS ^ag^ grade 3 or 4/20–60 mm (using VAS ^m^ 0–100 mm)/male and female	Cartistem^® af^	Surgical implantation/0.5 mL of the medicinal product per cm^2^ of cartilage lesion/12 months	

^a^ Body mass index, ^b^ Kellgren–Lawrence, ^c^ Western Ontario and McMaster Universities Osteoarthritis Index, ^d^ hyaluronan-based hydrogel suspended in hyaluronan solution, ^e^ triamcinolone acetonide, ^f^ hylan-based viscosupplement, ^g^ osteoarthritis, ^h^ intra-articular, ^i^ postinjection, ^j^ viscoelastic hydrogel for intra-articular use based on hyaluronic acid (HA) derivative, ^k^ randomized controlled trial, ^l^ phosphate buffered saline, ^m^ Visual Analog Scale, ^n^ hyaluronic acid, ^o^ intention-to-treat, ^p^ novel cross-linked hyaluronan, ^q^ soft gel-80, ^r^ cross-linked sodium hyaluronate containing triamcinolone hexacetonide, ^s^ cross-linked sodium hyaluronate viscosupplement, ^t^ transparent gel (viscosupplement) based on nonanimal stabilized hyaluronic acid (NASHA), ^u^ methylprednisolone acetate, ^v^ open-label extension phase, ^w^ noncross-linked animal-derived HA, ^x^ subcutaneous, ^y^ cross-linked hyaluronate, ^z^ linear high molecular weight hyaluronate, ^aa^ weight-bearing pain, ^ab^ polynucleotide sodium, ^ac^ 1,4-butanediol diglycidyl ether-cross-linked sodium hyaluronate, ^ad^ sodium hyaluronate, ^ae^ intra-articular hyaluronic acid, ^af^ medicinal product comprising culture-expanded allogeneic human umbilical-cord-blood-derived mesenchymal stem cells (hUCB-MSCs) and hyaluronic acid (HA) hydrogel, ^ag^ International Cartilage Repair Society, ^ah^ International Knee Documentation Committee.

## Data Availability

Not applicable.

## Supplementary Materials

The following supporting information can be downloaded at: https://www.mdpi.com/article/10.3390/polym14040839/s1, Figure S1: Chemical structures of polysaccharides, Figure S2: Chemical structures of proteins.
