# Supplementary figures and images for: Recent Developments in Hyaluronic Acid-Based Hydrogels for Cartilage Tissue Engineering Applications
Source: Polymers (Basel). 2022 Feb 21;14(4):839. doi: 10.3390/polym14040839 (PMC8963043; doi:10.3390/polym14040839)

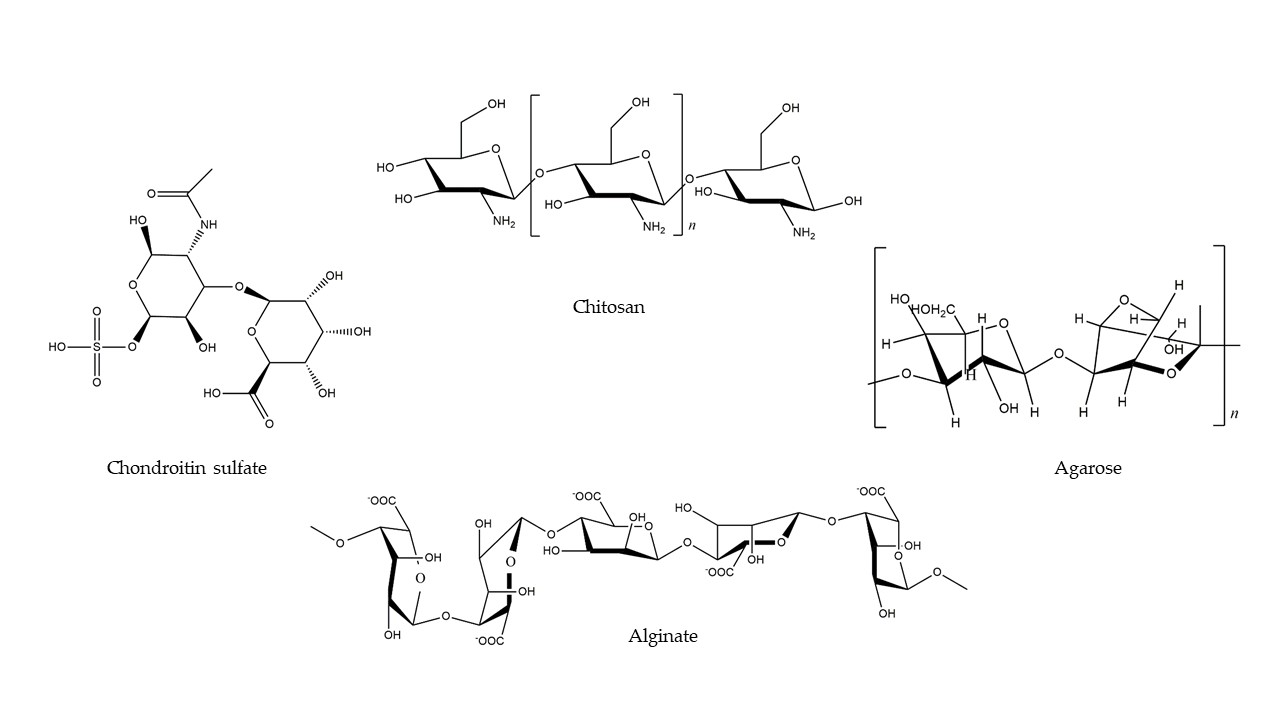

Supplement: Supplementary file 1 [file polymers-14-00839-s001.zip › S1.jpg]

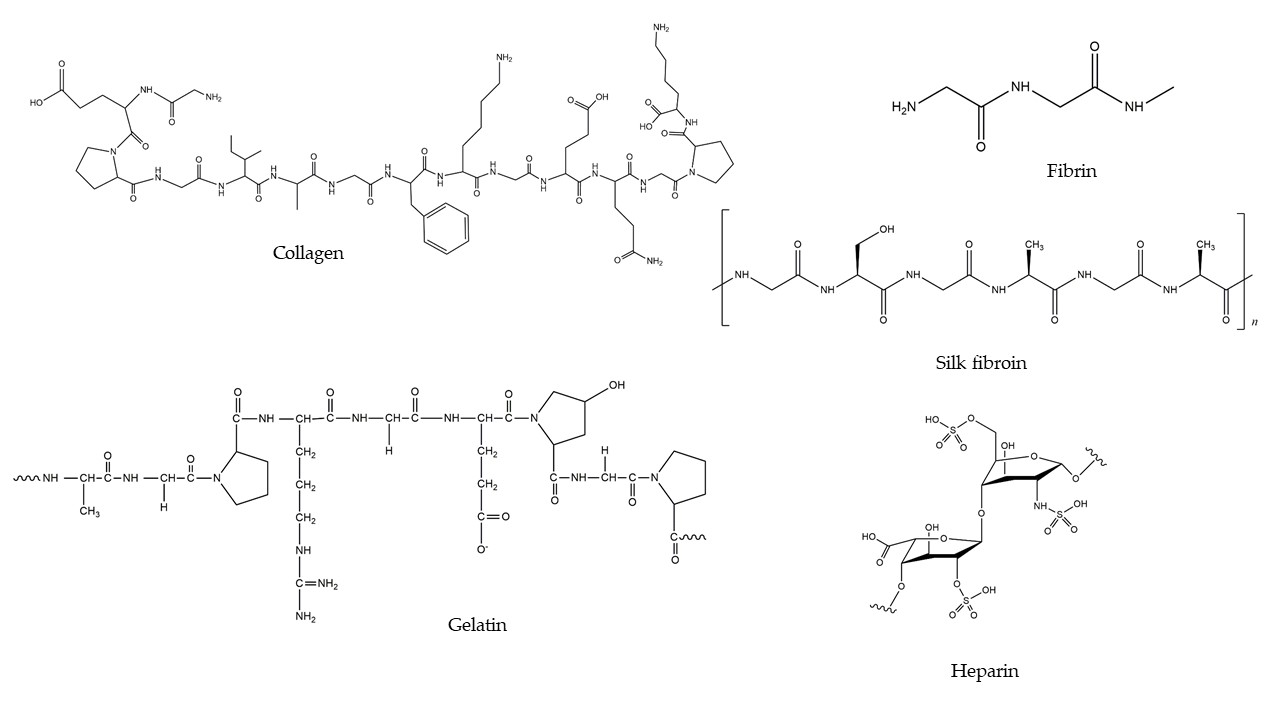

Supplement: Supplementary file 1 [file polymers-14-00839-s001.zip › S2.jpg]
